# Prevalence of inappropriate use behaviors of antibiotics and related factors among parents in eastern China: an online cross-sectional survey

**DOI:** 10.3389/fpubh.2025.1654293

**Published:** 2025-09-04

**Authors:** Huamin Sun, Zhenyu Wang, Jianping Huang, Na An, Man Jiao, Xinchen Zhu, Xinying Guo, Weiwei Tan, Weibin Zhang

**Affiliations:** ^1^Nantong Center for Disease Control and Prevention, Nantong, Jiangsu, China; ^2^The Second Affiliated Hospital of Nantong University, Nantong, Jiangsu, China

**Keywords:** antibiotic use, cognition, behavior, parental knowledge, self-medication

## Abstract

**Objective:**

This study aimed to investigate parental knowledge about antibiotic use, estimate the prevalence of inappropriate antibiotic use behaviors, identify related factors among parents in eastern China, and provide targeted suggestions for promoting rational antibiotic use in children.

**Methods:**

A cross-sectional survey was conducted between October and November 2024. A multistage stratified cluster random sampling method and a self-administered questionnaire were used to collect demographic and sociological characteristics, knowledge, and behaviors regarding antibiotic use from 2,575 parents in eastern China. Logistic regression models were used to analyze the factors influencing the inappropriate use of antibiotics.

**Results:**

Among the 2,575 participants, 49.05% reported self-storage of antibiotics (SSA) for their children, 21.90% reported self-medication with antibiotics (SMA), 57.13% reported non-adherence to antibiotic treatment (NAAT), and 8.04% reported using antibiotics for disease prevention in their children in the past year. Multivariate logistic regression analyses showed that parents scored less than 5 were more likely to report NAAT (adjusted OR [aOR] = 1.45, 95% CI = 1.15–1.83) and use antibiotics for disease prevention (aOR = 5.62, 95% CI = 3.26–9.69) in their children and less likely to report SSA (aOR = 0.68, 95% CI = 0.54–0.86). Storing antibiotics at home was associated with an increased likelihood of SMA (aOR = 7.25, 95% CI = 5.69–9.24) and using antibiotics for disease prevention in children (aOR = 8.42, 95% CI = 5.61–12.63).

**Conclusion:**

In Nantong City, eastern China, parents with low levels of antibiotic knowledge demonstrated a high frequency of inappropriate antibiotic use for children. To promote rational antibiotic use, efforts should focus on improving parental knowledge through health education, formulation of rational use strategies, and reducing antibiotic storage at home.

## 1 Introduction

The inappropriate use of antibiotics is a major contributor to the rise in antimicrobial resistance, worsening adverse drug reactions, and increased medical costs, which are becoming important global public health issues (1). Antibiotics are the most commonly used medications for children (2). Owing to the developmental stage of children, many organs and nervous system functions are not yet fully developed, and the risks associated with antibiotic use are greater than those in adults. Additionally, owing to their underdeveloped immune system, children are susceptible to illness and more likely to be exposed to unnecessary antibiotics, making the inappropriate use of antibiotics particularly prevalent in this population (3, 4). Therefore, it is necessary to determine whether antibiotics can be used in children.

Parents’ level of knowledge and attitude toward medication safety greatly impacts their children’s self-medication behavior. When children are sick, medication use depends primarily on their parents and guardians. Therefore, parents’ knowledge and behavior regarding antibiotic use greatly affect whether children can use antibiotics appropriately (5). If parents have misconceptions about antibiotics or engage in inappropriate behaviors in antibiotic use, the probability of children using antibiotics inappropriately will inevitably increase (6).

Self-medication with antibiotics (SMA), defined as the use of medicines based on personal experience without medical consultation (7), is one of the most common and inappropriate uses of antibiotics. A meta-analysis of 57 studies worldwide indicated that 24.0% of parents had administered antibiotics to their children without a doctor’s advice (4), and a cross-sectional study among 9,526 parents from three provinces of China indicated that the prevalence of SMA in children aged 0–13 years was 32.16% (8). Owing to differences in economic levels and antibiotic usage habits across different regions of China, there were significant variations in the prevalence of SMA among parents in different areas of China. A cross-sectional study conducted in two cities in China in 2020 showed that the incidence of SMA in children was 42.67% in Shaanxi Province and 18.78% in Zhejiang Province (9). Comparable trends have been observed internationally. For example, a recent study in central Iran reported a high prevalence of antibiotic use among children, underscoring the global scope of the issue (10).

In addition, self-storage with antibiotics (SSA), non-adherence to antibiotic treatment (NAAT), and the use of antibiotics for disease prevention by parents are important indicators of inappropriate use of antibiotics. Recently, there has been increasing research on the inappropriate use of antibiotics by Chinese parents; however, most studies have focused on SMA (11, 12). The inappropriate use behaviors of antibiotics among parents are diverse, but there have been no studies on the various inappropriate use behaviors and their influencing factors. Therefore, this study aimed to investigate parental knowledge about antibiotic use, assess the prevalence of inappropriate use behaviors, including SSA, SMA, NAAT, and the use of antibiotics for disease prevention, and identify related factors to provide targeted suggestions for promoting rational antibiotic use in children.

## 2 Materials and methods

### 2.1 Study population

Data for this study were obtained from a cross-sectional survey conducted in Nantong City, Jiangsu Province, eastern China, between October and November 2024. The survey covered all the counties in Nantong City. Nantong is one of the most developed cities in China. Using a stratified sampling method, we randomly selected two schools (one kindergarten and one primary school) from rural and urban areas in seven regions, respectively, followed by a random selection of the middle class from each kindergarten and two second-grade classes from each primary school. A minimum of 75 children was recruited from each school. This study was approved by the Institutional Ethics Committee of the Nantong Center for Disease Control and Prevention (reference number: 2024001), and the parents or guardians of the enrolled children signed consent forms. Overall, 2,710 participants were included in this study, and 2,575 valid questionnaires were obtained, culminating in an effective response rate of 95.02%.

We used the formula for a cross-sectional survey: *n* = *Z*^2^_1-*α*/2_ × *pq*/*d*^2^, when *α* = 0.05, *Z*_1-α/2_ = 1.96, *p* is the prevalence of the research target, *q* = 1-*p*, and *d* is the margin of error (*d* = 0.1 × *p*). In our study, *p* was the prevalence of inappropriate use of antibiotics among parents in the past year as a criterion, according to previous studies. The minimum sample size required to meet the research statistical efficiency was calculated to be 2,400; therefore, the sample size of our survey met the research requirements.

### 2.2 Data collection

Data were collected using China’s largest online survey platform (Questionnaire Star, https://www.wjx.cn). Participants accessed and completed the questionnaire via a QR (quick response) code using their smartphones. Before the investigation began, the researchers explained the completion requirements to kindergarten and elementary school teachers. The teachers distributed the QR codes and sent consent forms to the parents. Parents who agreed to participate signed the consent form and completed the online questionnaire. Two researchers independently verified the data and removed incomplete questionnaires to ensure the reliability of the survey data.

### 2.3 Study measurement

A self-administered questionnaire was used, consisting of three main sections: (1) Socio-demographic characteristics, including the parents’ age, education level, economic status, location of residence, medical background, children’s sex, school stage, and health status. (2) Parental knowledge of antibiotics. (3) Parental behaviors related to inappropriate antibiotic use in the past year. The following questions were included to assess inappropriate use: (1) Did you store antibiotics at home for future use? (2) Did you administer antibiotics to your children without a doctor’s prescription? (3) Did you immediately discontinue antibiotics once the child’s symptoms disappeared? (4) Did you encourage your children to take antibiotics prophylactically without medical advice?

### 2.4 Development of questionnaire

The questionnaire on antibiotic knowledge was developed based on a literature review (9, 13) and interviews with experts. Cronbach’s alpha for the antibiotic knowledge questionnaire was 0.726, indicating good internal consistency and reliability. Antibiotic knowledge was assessed by asking 10 questions about the role, use, and resistance of antibiotics. Response options were “true,” “false,” and “uncertain.” Correct answers were scored as 1, while incorrect or uncertain answers were scored as 0. The total score was calculated by summing the correct responses; a higher score indicated a higher level of antibiotic knowledge.

### 2.5 Statistical analysis

All statistical analyses were conducted using the IBM SPSS Statistics for Windows version 21.0 (IBM Corp., Armonk, NY, United States). Chi-square tests were used to compare the rates between the different groups. Logistic regression models were used to further explore the factors influencing inappropriate antibiotic use by controlling for sociodemographic variables. A two-sided *p* < 0.05 was considered statistically significant.

## 3 Results

### 3.1 Sociodemographic characteristics

As presented in Table 1, of the 2,575 valid participants, 1,357 (52.70%) were boys. The mean age of children was 6.04 ± 1.57 years. Among all the children, 46.83% were from kindergartens, and 51.26% were from urban places of residence. More than half of the respondents had a college or higher level of education (1,595, 61.94%). A small proportion (238; 9.24%) of the respondents had a medical background.

**Table 1:** Antibiotic knowledge score and influencing factors among parents of children in Nantong city.

Variable	AKS < 5 (*n* = 753)	AKS 5–7 (*n* = 1,183)	AKS > 7 (*n* = 639)	All (*n* = 2,575)	*χ* ^2^	*p*-value
Sex					0.419	0.811
Boys	395 (52.46)	631 (53.34)	331 (51.80)	1,357		
Girls	358 (47.54)	552 (46.66)	308 (48.20)	1,218		
School stages					1.163	0.559
Kindergarten	351 (46.61)	566 (47.84)	289 (45.23)	1,206		
Primary school	402 (53.39)	617 (52.16)	350 (54.77)	1,369		
Location					106.830^a^	<0.001
Urban	288 (38.25)	610 (51.56)	422 (66.04)	1,320		
Suburb or rural	465 (61.75)	573 (48.44)	217 (33.96)	1,255		
Only child					14.059^a^	<0.001
Yes	333 (44.22)	577 (48.77)	347 (54.30)	1,257		
No	420 (55.78)	606 (51.23)	292 (45.70)	1,318		
Self-perceived health status					10.422	0.034
Good	233 (30.94)	401 (33.90)	239 (37.40)	873		
Average	474 (62.95)	725 (61.28)	357 (55.87)	1,556		
Good	46 (6.11)	57 (4.82)	43 (6.73)	146		
Age of parents (years)					17.663	0.001
≤ 29	76 (10.09)	75 (6.34)	39 (6.10)	190		
30 ~ 44	644 (85.52)	1,074 (90.79)	585 (91.55)	2,303		
≥ 45	33 (4.38)	34 (2.87)	15 (2.35)	82		
Parents’ highest level of education					230.369^a^	<0.001
Middle school and below	222 (29.48)	188 (15.89)	37 (5.79)	447		
High school	211 (28.02)	240 (20.29)	82 (12.83)	533		
College and above	320 (42.50)	755 (63.82)	520 (81.83)	1,595		
Parents with medical background					104.979^a^	<0.001
Yes	19 (2.52)	100 (8.45)	119 (18.62)	238		
No	734 (97.48)	1,083 (91.55)	520 (81.38)	2,337		
Self-perceived economic status					133.555^a^	<0.001
Poor	459 (60.96)	495 (41.84)	189 (29.58)	1,143		
Average	274 (36.39)	630 (53.25)	406 (63.54)	1,310		
Good	20 (2.66)	58 (4.90)	44 (6.89)	122		
Elderly family members participated in children’s treatment					23.318^a^	<0.001
Yes	308 (40.90)	384 (32.46)	184 (28.79)	876		
No	445 (59.10)	799 (67.54)	455 (71.21)	1,699		

^aThe trend Chi square.^

### 3.2 Antibiotic knowledge among parents of children

The median antibiotic knowledge score (AKS) of parents was 6. Overall, 753 (29.24%) parents scored less than 5, 1,183 (45.94%) parents scored between 5–7, and 639 (24.82%) parents scored above 7. Higher AKS were observed among children lived in urban areas, were only children (all *p* < 0.001). Parental factors, such as higher educational level, medical background, higher household income, and where older family members were not involved in the children’s treatment were significantly associated with a higher AKS (all *p* < 0.001) (Table 1).

### 3.3 Prevalence of inappropriate use behaviors of antibiotics among parents of children

Among the 2,575 participants, 49.05% (1,263/2575) reported SSA at home, 21.90% (564/2575) had administered antibiotics to their children without a prescription in the past year. Most parents used antibiotics to treat their children without a prescription according to the leftover drug instructions (56.56%, 319/564), followed by previous doctor or pharmacist recommendations (32.80%, 185/564), and previous personal experience (10.64%, 60/564). β-lactams (76.42%, 431/564) were the most frequently type of antibiotics that parents self medicated, followed by macrolides (20.74%, 117/564) and other (2.84%, 16/564). The antibiotics that parents self medicate were cephalosporins (62.59%, 353/564), azithromycin (17.91%, 101/564), penicillins (13.83%, 78/564) and other (5.67%, 32/564). Among the 2,575 participants, 57.13% (1,471/2575) discontinued antibiotics treatment for children immediately after their symptoms disappeared, and 8.04% (207/2575) had taken antibiotics for disease prevention in the past year. Parents with a higher AKS were associated with an increased likelihood of storing antibiotics for children at home (*p* < 0.001). In contrast, parents with lower AKS were associated with an increased likelihood of discontinuing antibiotic treatment for their children immediately after the symptoms disappeared, and making them take antibiotics for disease prevention (all *p* < 0.05) (Table 2).

**Table 2:** Prevalence of inappropriate use behaviors of antibiotics among parents of children.

Variable	AKS < 5 (*n* = 753)	AKS 5–7 (*n* = 1,183)	AKS > 7 (*n* = 639)	All (*n* = 2,575)	*χ* ^2^	*p*-value
Self-storage with antibiotics					28.217^a^	<0.001
Yes	306 (40.64)	608 (51.39)	349 (54.62)	1,263		
No	447 (59.36)	575 (48.61)	290 (45.38)	1,312		
Self-medication with antibiotics					4.959	0.084
Yes	144 (19.12)	276 (23.33)	144 (22.54)	564		
No	609 (80.88)	907 (76.67)	495 (77.46)	2011		
Non-adherence to antibiotic treatment					8.862^a^	0.003
Yes	452 (60.03)	687 (58.07)	332 (51.96)	1,471		
No	301 (39.97)	496 (41.93)	307 (48.04)	1,104		
Used antibiotics prophylactically					40.983^a^	<0.001
Yes	93 (12.35)	95 (8.03)	19 (2.97)	207		
No	660 (87.65)	1,088 (91.97)	620 (97.03)	2,368		

^aThe trend Chi square.^

### 3.4 Factors associated with inappropriate use behaviors of antibiotics among parents of children

Multivariate logistic regression analyses showed that parents were more likely to store antibiotics at home if their children had brothers or sisters (adjusted OR [aOR] = 1.20, 95% CI = 1.02–1.41), had poor health status (aOR = 1.84, 95% CI = 1.28–2.66), and if the parents had a high school education (aOR = 1.61, 95% CI = 1.24–2.10), a college education and above (aOR = 1.87, 95% CI = 1.46–2.38), or medical background (aOR = 1.85, 95% CI = 1.39–2.47). In contrast, parents with AKS < 5 (aOR = 0.68, 95% CI = 0.54–0.86) were associated with an decreased likelihood of self-storage of antibiotics at home.

Children who had brothers or sisters (aOR = 1.33, 95% CI = 1.08–1.64), had average health status (aOR = 1.44, 95% CI = 1.15–1.81) or poor health status (aOR = 3.71, 95% CI = 2.46–5.60), and whose parents had a high school education (aOR = 1.68, 95% CI = 1.17–2.42), college education and above (aOR = 1.70, 95% CI = 1.21–2.40), or stored antibiotics at home for use (aOR = 7.25, 95% CI = 5.69–9.24) were associated with an increased likelihood of self-medication behavior.

Children with poor health status (aOR = 2.78, 95% CI = 1.83–4.21) and parents with AKS < 5 (aOR = 1.45, 95% CI = 1.15–1.83) or AKS 5–7 (aOR = 1.33, 95% CI = 1.09–1.62) were associated with an increased likelihood of discontinuing antibiotic treatment for children immediately after the symptoms disappear. There was a dose–response relationship between AKS and NAAT. Furthermore, parents with AKS < 5 (aOR = 5.62, 95% CI = 3.26–9.69) or AKS 5–7 (aOR = 2.86, 95% CI = 1.70–4.80) and those who would store antibiotics at home (aOR = 8.42, 95% CI = 5.61–12.63) were associated with an increased likelihood of administering antibiotics to their children for disease prevention. There was a dose–response relationship between AKS and administering antibiotics to their children for disease prevention (Table 3).

**Table 3:** Multivariate logistic regression analysis of self-storage with antibiotics, self-medication with antibiotics, non-adherence to antibiotic treatment, and used antibiotics prophylactically.

Variable	Self-storage with antibiotics aOR (95% CI)	Self-medication with antibiotics aOR (95% CI)	Non-adherence to antibiotic treatment aOR (95% CI)	Used antibiotics prophylactically aOR (95% CI)
Sex (ref boys)				
Girls	0.98 (0.84–1.15)	0.97 (0.79–1.19)	1.04 (0.89–1.22)	0.96 (0.71–1.30)
School stages (ref kindergarten)				
Primary school	0.98 (0.83–1.15)	0.86 (0.70–1.06)	1.05 (0.89–1.23)	1.09 (0.80–1.47)
Location (ref urban)				
Suburb or rural	0.88 (0.74–1.04)	0.81 (0.65–1.02)	1.00 (0.84–1.19)	0.85 (0.61–1.19)
Only child (ref yes)				
No	1.20 (1.02–1.41)^*^	1.33 (1.08–1.64)^**^	1.12 (0.95–1.32)	1.10 (0.80–1.50)
Self-perceived health status (ref good)				
Average	1.03 (0.87–1.22)	1.44 (1.15–1.81)^**^	0.96 (0.81–1.13)	0.77 (0.56–1.11)
Poor	1.84 (1.28–2.66)^**^	3.71 (2.46–5.60)^***^	2.78 (1.83–4.21)^***^	0.94 (0.51–1.73)
Age of parents (years) (ref ≤ 29)				
30 ~ 44	0.97 (0.71–1.32)	1.06 (0.71–1.60)	1.26 (0.93–1.71)	1.52 (0.80–2.87)
≥ 45	1.32 (0.77–2.27)	0.72 (0.34–1.49)	1.46 (0.85–2.50)	1.97 (0.77–5.06)
Parents’ highest level of education (ref middle school and below)				
High school	1.61 (1.24–2.10)^***^	1.68 (1.17–2.42)^**^	1.23 (0.95–1.59)	1.11 (0.71–1.74)
College and above	1.87 (1.46–2.38)^***^	1.70 (1.21–2.40)^**^	1.26 (0.99–1.61)	0.78 (0.50–1.22)
Parents with medical background (ref no)				
Yes	1.85 (1.39–2.47)^***^	1.21 (0.87–1.69)	1.04 (0.78–1.37)	0.79 (0.43–1.45)
Self-perceived economic status (ref poor)				
Average	1.03 (0.87–1.23)	0.95 (0.76–1.19)	0.93 (0.78–1.10)	1.23 (0.89–1.70)
Good	0.85 (0.58–1.26)	1.17 (0.71–1.93)	0.77 (0.52–1.13)	1.07 (0.46–2.49)
Elderly family members participated in children’s treatment (ref no)				
Yes	1.18 (0.99–1.39)	1.19 (0.96–1.48)	1.03 (0.87–1.23)	1.09 (0.80–1.48)
AKS (Ref AKS > 7)				
AKS < 5	0.68 (0.54–0.86)^**^	1.04 (0.76–1.41)	1.45 (1.15–1.83)^**^	5.62 (3.26–9.69)^***^
AKS 5–7	0.98 (0.80–1.19)	1.11 (0.86–1.44)	1.33 (1.09–1.62)^**^	2.86 (1.70–4.80)^***^
Self-storage with antibiotics (ref no)				
Yes	–	7.25 (5.69–9.24)^***^	1.02 (0.87–1.19)	8.42 (5.61–12.63)^***^

*P < 0.05, **p < 0.01, ***P < 0.001.

aOR, adjusted odds ratio; Ref, reference group.

## 4 Discussion

This study investigated knowledge about antibiotic use and inappropriate use behaviors of antibiotics and explored their related factors among parents in eastern China. We conducted a survey of parents from one kindergarten and one primary school in rural and urban areas of each region in Nantong City, which ensured that the sample was representative.

Among the parents, 29.24% scored <5, indicating a low awareness rate of antibiotic use knowledge among parents in Nantong City. Parents with low awareness of antibiotic use have insufficient understanding of the dangers of antibiotic abuse, lack the ability to use antibiotics reasonably, and are susceptible to inappropriate antibiotic use (14). This study suggests that children lived in rural areas, had brother or sister have lower levels of antibiotic awareness. Parents with lower education, without medical backgrounds, lower income, where older family members were involved in the children’s treatment with a lower AKS. Health education on the rational use of antibiotics should be emphasized to these parents to improve their understanding of the dangers associated with inappropriate antibiotic use in children. Engaging local health volunteers may also be a practical approach, as shown in Iran during the COVID-19 pandemic, where community volunteers significantly improved public health literacy and preventive behaviors (15).

Our study found that the prevalence of self-storage of antibiotics at home for children was 49.05% in the past year. Additionally, the research results showed that SSA was a risk factor for SMA by parents (aOR = 7.25, 95% CI = 5.69–9.24) and for administering antibiotics to children for disease prevention (aOR = 8.42, 95% CI = 5.61–12.63), which is consistent with previous research results (16, 17). A previous study showed that the antibiotics stored at home come from two main sources: leftover prescription drugs and purchases from pharmacies (18). Chinese parents can easily obtain over-the-counter antibiotics. Studies have shown that Chinese parents can still purchase antibiotics from retail pharmacies without a prescription (19, 20), which increases the opportunity for parents to store antibiotics. When children experienced similar symptoms as before or when they were sick, parents used antibiotics stored or remaining at home to treat the child based on past experience. This study suggests that parents with a medical background are more likely to keep antibiotics at home. Conversely, parents with a medical background were more likely to obtain antibiotics; however, parents with a medical background had a higher cognitive score for antibiotics. They believed that they had the medical knowledge and ability to provide reasonable medications to their children.

Our study found that the prevalence of SMA was 21.90%, which was lower than that of SMA in Mexican children (49.6%) (21), but higher than the prevalence of SMA in children in Europe (8%) (4), Zhejiang Province (18.78%), and China (9). SMA by parents promotes the excessive use of antibiotics in children, which might lead to a series of adverse consequences, such as misuse and abuse of antibiotics, concealment of true illness, and delayed treatment.

Our study suggests that 57.13% of parents discontinued with antibiotic treatment immediately after their children’s symptoms disappeared. Owing to inconsistent criteria for determining NAAT, it was not possible to compare the prevalence of NAAT in this study with that in previous studies. The reason why parents did not follow antibiotic treatment might be that Chinese parents are concerned about the side effects of antibiotics on their children when using them (22). Discontinuing antibiotics in advance could weaken the therapeutic effect and lead to incomplete sterilization; however, this could lead to residual antibiotics at home, thereby increasing the risk of SMA in children.

This study found that parents with lower antibiotic awareness scores had a greater risk of NAAT and took antibiotics for disease prevention, whereas parents with higher awareness scores were more likely to store antibiotics at home. Improving parents’ awareness of antibiotics could reduce the risk of NAAT and taking antibiotics for disease prevention; however, it might increase the risk of parents storing antibiotics at home, thereby increasing the inappropriate use of antibiotics. Other studies have shown that parents have higher antibiotic awareness scores and are more inclined to use antibiotics on their own (5). The research results suggest that the relationship between parental antibiotic awareness and the reasonable use of antibiotics was complex, and simply improving parental antibiotic awareness might have counterproductive effects (23). Similar complexity has been noted in other public health domains such as COVID-19, where population-level studies have demonstrated that knowledge alone does not always translate into safe practices without addressing underlying attitudes and contextual factors (24). Further research and exploration should simultaneously reduce parents’ inappropriate use of antibiotics while improving their awareness.

Our study also suggested that parents in poor physical condition were more likely to engage in inappropriate use behaviors, such as SSA, SMA, and NAAT, which is consistent with previous research findings (13). Children with poor health require frequent use of medication. Long-term medication habits make parents more dependent on antibiotics, increasing the likelihood of storing antibiotics at home and thus increasing the risk of inappropriate use of antibiotics.

This study has some limitations. First, this was a cross-sectional study, and it is difficult to clarify the causal relationship or the direction between the inappropriate use of antibiotics by parents and related influencing factors. For example, while it finds that lower knowledge is associated with non-adherence, we cannot be sure if poor knowledge leads to non-adherence or if experiencing antibiotic use (or misuse) influences how parents score on knowledge questions. Further prospective studies are required to validate the conclusions of this study. Second, using a self-administered questionnaire to investigate the antibiotic use behavior of parents of children within 1 year. Self-reported recall over 1 year is subject to social desirability and recall bias, which is particularly acute for sensitive health behaviors. The results were inevitably subject to recall and social desirability bias. Third, this study was only conducted in one prefecture-level city in eastern China, and the socioeconomic development level of Nantong City is relatively high in eastern China. Therefore, the results do not represent other regions in eastern China.

Parents in eastern China had lower antibiotic awareness scores. It is necessary to raise parental awareness of the dangers associated with inappropriate antibiotic use in children and strengthen their awareness of the rational use of antibiotics. Inappropriate use of antibiotics by parents is common, and it is necessary to develop rational medication strategies, limit the outflow of over-the-counter antibiotics, and reduce household antibiotic reserves and inappropriate use behaviors.

## Data Availability

The original contributions presented in the study are included in the article/Supplementary material, further inquiries can be directed to the corresponding authors.
