# Supplementary material for: Prevalence of inappropriate use behaviors of antibiotics and related factors among parents in eastern China: an online cross-sectional survey
Source: Front Public Health. 2025 Sep 4;13:1654293. doi: 10.3389/fpubh.2025.1654293 (PMC12443743; doi:10.3389/fpubh.2025.1654293)
Supplement: Supplementary file 1 [file Table_1.DOCX]

Supplementary Material

**Table S1. Response to knowledge on antibiotic use among parents of children**

| Items | Number of incorrect response | Percentage (%) |
| --- | --- | --- |
| 1.Antibiotics can treat viral infections. | 1668 | 64.78 |
| 2.Antibiotics are the same as anti-inflammatory agents. | 1845 | 71.65 |
| 3.The more expensive the antibiotic, the effectiveness may necessarily be better. | 1043 | 40.50 |
| 4.Using multiple antibiotics is necessarily better than using only one. | 619 | 24.04 |
| 5.Using antibiotics can speed up your child’s cold recovery. | 1050 | 40.78 |
| 6.Do you think antibiotics are appropriate when your child has the following conditions (diseases)? | 2065 | 80.19 |
| 7.Do you know which of the following antibiotics are not suitable for children to use? | 2107 | 81.83 |
| 8.If your child needs antibiotics, it is better to be administered by infusion. | 980 | 38.06 |
| 9.Excessive use of antibiotics is a serious problem in China. | 554 | 21.51 |
| 10.Antibiotic overuse can result in antibiotic resistance. | 334 | 12.97 |

**
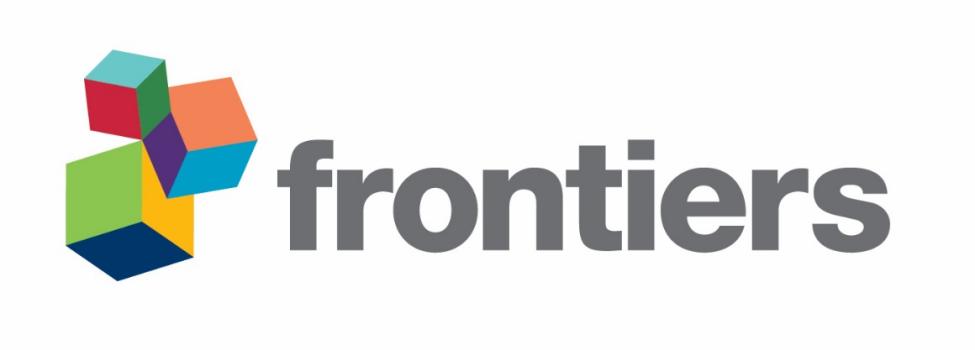
**
